# Neuroinflammation in mouse models of Alzheimer's disease

**DOI:** 10.1111/cen3.12475

**Published:** 2018-09-23

**Authors:** Takashi Saito, Takaomi C. Saido

**Affiliations:** ^1^ RIKEN Center for Brain Science Laboratory for Proteolytic Neuroscience Wako Japan; ^2^ Department of Neuroscience and Pathobiology Research Institute of Environmental Medicine Nagoya University Wako Japan

**Keywords:** Alzheimer's disease, glial cell, mouse model, neuroimmune communication, neuroinflammation

## Abstract

Alzheimer's disease (AD) is the most common type of neurocognitive disorder. Although both amyloid β peptide deposition and neurofibrillary tangle formation in the AD brain have been established as pathological hallmarks of the disease, many other factors contribute in a complex manner to the pathogenesis of AD before clinical symptoms of the disease become apparent. Longitudinal pathophysiological processes cause patients’ brains to exist in a state of chronic neuroinflammation, with glial cells acting as key regulators of the neuroinflammatory state. However, the detailed molecular and cellular mechanisms of glial function underlying AD pathogenesis remain elusive. Furthermore, recent studies have shown that peripheral inflammatory conditions affect glial cells in the brain through a process of neuroimmune communication. Such disease complexities make it difficult for the pathogenesis of AD to be understood, and impede the development of effective therapeutic strategies to combat the disease. Relevant AD animal models are thus likely to serve as a key resource to overcome many of these issues. Furthermore, as the pathogenesis of AD might be linked to conditions both within the brain as well as peripherally, it might become necessary for AD to be studied as a whole‐body disorder. The present review aimed to summarize insights regarding current AD research, and share perspectives for understanding glial function in the context of the pathogenesis of AD.

## Introduction

Neurodegenerative disorders, such as Parkinson's disease, amyotrophic lateral sclerosis, Huntington's disease and Alzheimer's disease (AD), are proving to be the most difficult diseases to prevent or treat, and remain as unmet medical needs. AD is the primary cause of neurocognitive disorders in the elderly, and poses a huge socioeconomic burden for modern society. The number of patients with dementia is estimated to be >46 million people worldwide, and is increasing unabated each year. To the present time, only some symptomatic treatments have been found to be effective. To overcome this impasse and to develop effective treatments, elucidation of the molecular and cellular mechanisms underlying the pathogenesis of AD with a view to identifying druggable targets must be a priority.

**Figure 1:**
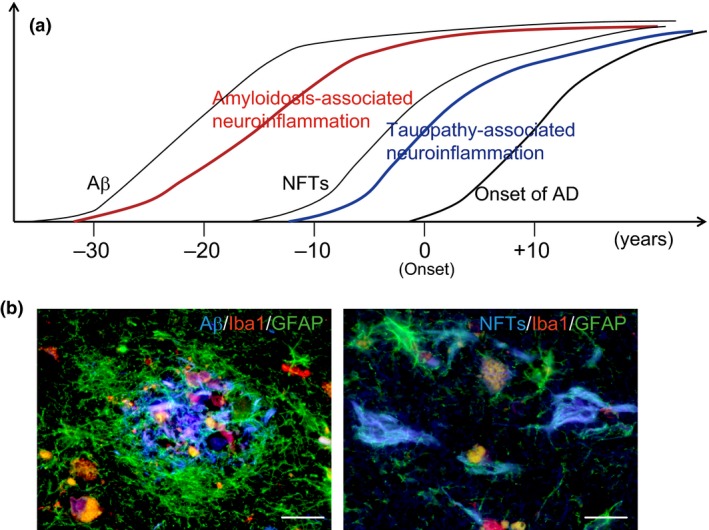
(a) Time‐course of Alzheimer's disease (AD) progression. Amyloid β peptide (Aβ) deposition begins >25 years before the onset of AD and is followed by neurofibrillary tangles (NFT) formation. This leads to neurodegeneration and neuronal cell death. Both amyloid‐associated and tauopathy‐associated neuroinflammation might facilitate AD pathogenesis. (b) Immunohistochemical staining of gliosis in the human AD brain. 1‐Fluoro‐2,5‐bis(3‐carboxy‐4‐hydroxystyryl)benzene (blue fluorescence) binds to β‐sheet structures, such as dense‐cored Aβ plaques (left panel) and NFTs (right panel), respectively, with ionized calcium binding adaptor molecule 1 (Iba1)‐positive microgliosis shown in red and glial fibrillary acid protein (GFAP)‐positive astrocytosis in green. Scale bar, 20 μm.

Senile plaques composed of extracellular amyloid β peptide (Aβ) and neurofibrillary tangles (NFT), which are the aggregates of intracellular hyperphosphorylated tau protein, are hallmarks of the AD brain.^1^ Aβ deposition and NFT formation in the cortical region of the brain begin appearing 25–30 years and 15 years, respectively, before the clinical onset of AD (Fig. 1a).^2^ Aβ is generated proteolytically from amyloid precursor protein (APP) to subsequently form oligomeric Aβ, which aggregates into senile plaques. Although microtubule‐associated protein tau stabilizes microtubules in the axon, pathological tau mislocalizes through an unknown mechanism and forms NFT aggregates in the neuronal dendrites and cell body. These protein aggregates in the brain environment induce the activation of microglia and astrocytes, which results in microgliosis and astrocytosis around the pathological structures.

Glial cells are thus chronically activated in the brain before the onset of AD,^3^ with the associated chronic inflammation contributing to the pathogenesis of AD. In the AD brain, microgliosis and astrocytosis as a consequence of the presence of senile plaques and NFTs can be detected immunohistochemically, with these glial cells showing a pathology‐specific morphology (Fig. 1b). Although the extent of gliosis is correlated with cortical thickness and neurodegeneration, the roles of different glial cells in neurodegenerative processes remain unclear.^4^ Relevant animal models are required for these processes to be investigated in greater detail.

## Animal models for AD and neuroinflammation

**Figure 2:**
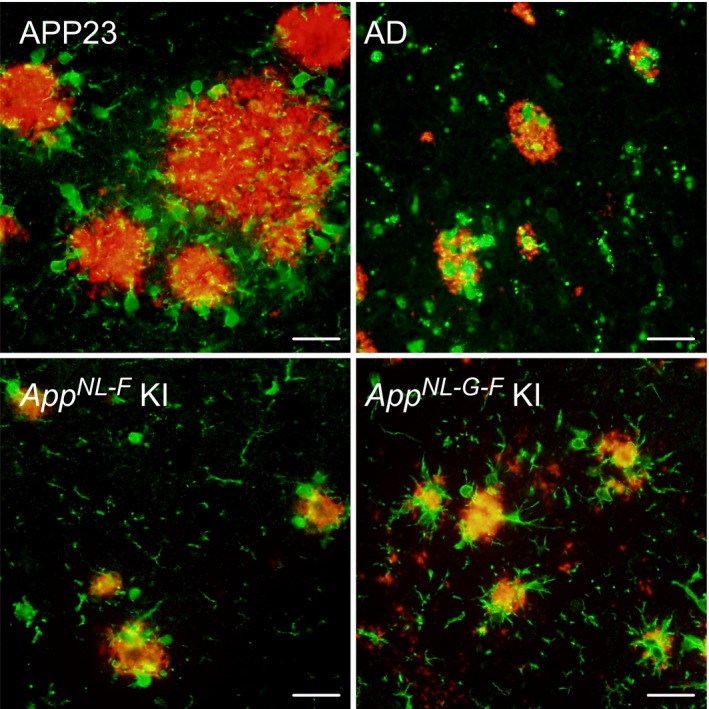
Pathological differences in amyloidosis between mouse models and human brain tissue. Double staining for Aβ (anti‐Aβ antibody: 82E1, red) and ionized calcium binding adaptor molecule 1‐positive microgliosis (green) was carried out using brain sections obtained from amyloid precursor protein (APP)23 mice, *App* knock‐in mice and post‐mortem brain tissue from an Alzheimer's disease (AD) patient. Scale bar, 25 μm. F, Iberian mutation; G, Arctic mutation; NL, Swedish mutation.

Animal models representing relevant pathologies with as few artifactual anomalies as possible are necessary. To this end, a number of AD mouse models have been developed,^5^ with APP overexpressing mice, such as APP transgenic (APP Tg) mice, having been used widely,^6^, ^7^ although they are associated with considerable technical and physiological issues. For example, amyloid plaques in some APP Tg mice, particularly Tg2576 and APP23 mice, were found to be very large in size and composed mainly of Aβ40,^8^ making the plaques decidedly different from those seen in AD patients (Fig. 2). These findings were due to technical limitations associated with the animal models, which were based on an APP overexpression paradigm. To overcome these drawbacks, we created two strains of *App* knock‐in (KI) mice,^9^ named *App* ^*NL‐F*^ KI and *App* ^*NL‐G‐F*^ KI. *App* ^*NL‐F*^ KI mice harbor the Swedish mutation (NL) and the Iberian mutation (F), whereas *App* ^*NL‐G‐F*^ KI mice also harbor the Arctic mutation (G). Both *App* KI mouse strains showed relevant amyloid deposition composed of pathological Aβ42, similar to that in AD patients (Fig. 2).^9^ Advantages associated with using the *App* KI strains have been described,^10^, ^11^ with these mouse strains showing fewer artifactual anomalies compared with APP overexpressing mice.^12^, ^13^ However, we did not observe NFT in the *App* KI mice during their lifespan, suggesting that the mice might also be useful as preclinical AD mouse models to investigate the pathological role of amyloidosis and amyloid‐associated neuroinflammation. Hama et al. succeeded in using 3‐D visualization of resting and activated microglia in the brains of *App* KI mice and AD patients using an optical clearing technique, and showed that microglia are frequently associated with diffuse plaques in the AD brain.^14^ Zhang et al. further reported that *App* KI mice show mushroom spine loss,^15^ which could reflect microglia‐mediated synapse loss in AD.^16^ Furthermore, Castillo et al. reported amyloidosis‐dependent transcriptomic profiles in 3xTg AD mice^17^ and *App* ^*NL‐G‐F*^ KI mice.^18^ In contrast to 3xTg AD mice, *App* ^*NL‐G‐F*^ KI mice express genes in common with AD patients, such as neuroinflammation‐related genes (*C4a/C4b*,* Cd74*,* Ctss*,* Gfap*,* Nfe212*,* Phyhd1*,* S100b*,* Tf*,* Tgfbr2* and *Vim*) and AD risk factor genes (*Abi3*,* Apoe*,* Bin2*,* Cd33*,* Ctsc*,* Dock2*,* Fcer1g*,* Frmd6*,* Hck*,* Inpp5D*,* Ly86*,* Plcg2*,* Trem2* and *Tyrobp*).^18^ Thus, *App* KI mice might overcome some of the previous limitations associated with APP overexpression‐based mouse models, and could thus serve as a useful research tool for further investigations.

Various tauopathy mouse models have also been generated,^19^ with most tau transgenic models harboring tau mutations known to be associated with frontotemporal dementia with parkinsonism linked to chromosome 17 (FTDP‐17), and not associated with AD.^20^ The mutations accelerate the self‐aggregation of tau or give rise to an isoform shift from 3‐repeat to 4‐repeat tau. In any case, tau transgenic mice containing FTDP‐17 mutations form NFT without amyloid plaque deposition, meaning that these mouse models might contribute to elucidation of the role of tauopathy‐associated neuroinflammation. The microglial phenotype changes from a ramified type to an amoeboid type during the development of tauopathy in rTg4510 mice.^21^ P301S‐tau Tg mice show reactive gliosis before tau aggregation, whereas immunosuppression of P301S‐tau Tg mice with FK506 attenuates tau pathology.^22^ In another scenario, amyloid deposition exacerbates NFT formation in JNPL3 transgenic mice.^23^ Although the pathomolecular mechanisms linking Aβ deposition to NFT formation, or NFT formation to neurodegeneration and neuronal cell death remain unclear, these outcomes suggest that neuroinflammation could link amyloid pathology and NFT formation as an important pathophysiological event in the development of AD. To further elucidate a pathological role of neuroinflammation in AD, different strategies using immune challenge‐based models and neurotoxin‐induced AD models have been used.^24^ In virtually all scenarios considered, animal models will be indispensable for elucidating the molecular and cellular mechanisms of AD pathogenesis, and for developing effective strategies to prevent and treat the disease.

## Neuroinflammatory glial responses (relevance to the brain's microenvironment)

Microglia, the principal innate immune cells in the brain, carry out macrophage‐like phagocytic actions to remove pathogens and to protect neurons from toxic species. However, microglia produce and release molecules, such as reactive oxygen species and nitric oxide, that are neurotoxic.^25^ They also generate pro‐inflammatory cytokines and chemokines in response to danger signals, and communicate with astrocytes.^26^ To this end, the dysregulation of microglial activity has been associated with AD pathogenesis in the aged brain.^27^ Recently, it was reported that TREM2, as well as CD36 and the receptor for advanced glycation end‐products, work as Aβ sensor molecules and activate microglia.^28^ A consistent and elevated expression of TREM2 was described in *App* ^*NL‐G‐F*^ KI mice.^18^ Activated microglia also produce the pro‐inflammatory cytokines CCL3/MIP‐1α and interleukin (IL)‐6,^28^ the latter being a key component of the senescence‐associated secretory phenotype,^29^ which might provide a pathophysiological connection between cellular/tissue senescence and age‐related chronic diseases in the brain, including AD. Furthermore, pro‐inflammatory gene polymorphisms, including CCL3/MIP‐1α and IL‐6, have been identified as risk factors for AD.^30^

The genetic modulation of inflammation‐related factors has been investigated in various transgenic models of AD.^31^ These studies commonly showed that the modulation of inflammatory factors alters amyloid pathology and tau phosphorylation in the mouse models used. Interestingly, the inflammasome, a key inflammatory signaling platform in immune cells that activates IL‐1β and IL‐18 through NLRP3/ASC/Caspase1 activation,^32^ in microglia might contribute to AD pathogenesis in APP/PS1 mice.^33^, ^34^ To this end, microglia‐derived ASC (a constituent of the inflammasome) has been shown to regulate amyloidosis in APPswe/PSEN1dE9 mice.^35^ Modulation of glial function through the manipulation of cytokines/chemokines and their receptors has also been investigated.^31^, ^36^ These studies provided evidence that the microglial fractalkine receptor (CX3CR1) could potentially exacerbate tau pathology and neuronal cell death,^37^, ^38^ and that microglia also expand tau propagation through the exosome.^27^, ^39^ These findings support the notion that reactive microglial neuroinflammation accelerates AD pathogenesis, particularly by linking tau pathology with neurodegeneration.

Astrocytes serve multiple functions, including providing support to endothelial cells that form neurovascular units in the blood–brain barrier, supplying nutrients to the central nervous system, maintaining the extracellular balance of electrolytes and water, and repairing or remodeling tissue during the process of traumatic brain injury or neuroinflammation. As shown in Figure 1b, reactive astrocytes can be typically observed in the vicinity of amyloid plaque (plaque‐associated astrocytes). Although the primary function of the plaque‐associated astrocytic response remains unclear, deletion of the glial filament proteins glial fibrillary acid protein and vimentin in APP/PS1 mice increased the number of dystrophic neurites,^40^, ^41^ whereas astrocyte‐producing kallikrein‐related peptidase 7 contributed to Aβ degradation.^42^, ^43^ Astrocytes also contribute to the clearance of Aβ and other debris from the brain through astrocytic transport, the so‐called “glymphatic system”.^44^ As the glymphatic system is comparable with the lymphatic system in peripheral organs, astrocytes might act as a gateway from the brain to the blood vessels. Recently, phagocytic astrocytes were observed at ischemic sites of the brain.^45^ Astrocytic phagocytosis has been suggested to engulf and degrade plaque‐associated synaptic dystrophies in APP/PS‐1 mice and AD brain.^46^ Furthermore, neurotoxic A1 astrocytes are induced by activated microglia;^47^ to this end, a glucagon‐like peptide‐1 receptor agonist was postulated to act as a potential neuroprotective agent through the suppression of A1 astrocytes in a mouse model of Parkinson's disease.^48^ Although further classification of astrocyte cell types is required, plaque‐associated reactive astrocytes could protect neurons surrounding amyloid plaques in the early stages of AD pathogenesis. While these glial cells thus serve as “guardians” of the brain microenvironment, any dysregulation of glial communication could lead to a neurotoxic state.

The receptivity and responsiveness of glial cells are different with respect to amyloid plaques compared with NFT in the AD brain. Plaque‐associated astrocytes surround amyloid plaques, whereas microglia attack the inside of the plaques. However, microglia are unable to reach the inside of amyloid plaques in APP23 mouse brains due to the unphysiologically large size of these plaques (Fig. 2). Consequently, the microglial response in APP Tg mice is likely to be different from that in *App* KI mice and in the AD brain. The morphological pattern of reactive astrocytes and microglia in response to FSB‐positive NFT (ghost tangles) was different from that in response to Aβ deposition (Fig. 1b). Regulation of the glial response might therefore change markedly depending on the pathological stage of tauopathy.^21^, ^49^ However, it is still unclear which glial cells, astrocytes or microglia, recognize these abnormal protein aggregates as danger signals, and how this is achieved, especially considering that the communication between astrocytes and microglia is regulated by pathology‐associated cytokines/chemokines.

In summary of the above literature, the cytokines induced by amyloidosis are different from those induced by tauopathy in the different mouse models, meaning that it might be possible to distinguish amyloidosis‐associated glial responses (neuroinflammation) from tauopathy‐associated neuroinflammation, as shown in Figure 1a.^18^, ^31^, ^36^, ^37^, ^38^ Indeed, in a manner similar to that describing amyloidosis and tauopathy in the AD brain, glial pathology in amyloidosis mouse models is different from that in tauopathy mouse models. Furthermore, at the stage when both amyloid and tau pathologies exist, dysregulation of glial communication in the brain microenvironment along with the long‐lasting abnormal activation of glial cells might facilitate the pathogenesis of AD.

## Neuroinflammation and the interaction between brain and peripheral tissue (whole‐body macroenvironment)

Evidence from epidemiological studies pointed to a possible link between the use of non‐steroidal anti‐inflammatory drugs (NSAIDs) and a decreased risk of AD in people with rheumatoid arthritis.^50^ Subsequently, the long‐term use of NSAIDs was found to potentially protect individuals against AD, but not against vascular dementia.^51^ A number of studies using different AD mouse models also suggested that NSAIDs improve Aβ‐mediated brain dysfunction. Although the protective mechanisms by which NSAIDs exert their effects remain unclear, inflammation in the brain and/or periphery could be involved in the pathogenesis of AD. In contrast to the epidemiological evidence, a recent meta‐analysis on the effects of NSAID treatment reported no beneficial effect on AD.^52^ These conflicting results suggest that NSAIDs do not improve AD pathogenesis directly in the brain, but that systemic inflammation, such as that seen with rheumatoid arthritis, might affect the brain pathologically. Recent evidence also suggests that inflammatory diseases, such as osteoporosis,^53^ diabetes,^54^ cancer^55^ and infection,^56^ are possibly implicated in brain disorders, and that these diseases could affect brain function through immune responses elicited in the periphery; that is, through neuroimmune communication. Furthermore, treatment strategies against such diseases might influence brain function and the macroenvironment, as reported for cancer‐related cognitive impairment^57^ or HIV‐associated neurocognitive disorder,^58^ possibly leading to the onset of brain disorders. Recently, accumulating evidence has suggested a role of peripheral immune cells, particularly CD4^+^ T cells, in the central nervous system. CD4^+^ T cells have been suggested to affect the activation of microglia, and to alter the Aβ burden in APP Tg mice and AD patients.^59^, ^60^, ^61^ Thus, future investigations need to clarify the role of both tissue resident immune cells and circulating immune cells in the pathogenesis of AD.

Interestingly, gut microbiota and associated metabolites have been described to influence brain dysfunction^62^, ^63^ and to modulate the host immune system.^64^ This “gut–brain axis” has consequently received attention in several research fields. While it was reported that factors, such as obesity,^65^ exercise,^65^ diet/nutrition,^65^ circadian rhythm,^66^ sleep,^67^ stress^68^ and aging,^69^ modulate gut microbiotic conditions, such factors might also affect brain function and brain disorders. The body's macroenvironment, particularly the role played by inflammatory factors and immune cells including microglia and astrocytes, almost certainly contributes to the correct physiological functioning of the brain, as well as to the pathogenesis of brain disorders when this environment is disturbed. Taken together, these observations shed new light on the notion that the pathogenesis of AD might be linked not only to conditions within the central nervous system, but also to peripheral conditions, thus making it a whole‐body disorder. To further investigate such whole‐body interactions, studies using relevant animal models and the *in vivo* imaging of neuroinflammation will be critical to understanding the mechanisms underlying AD and to predicting therapeutic outcomes.^70^

## Conclusion

Numerous AD studies showing the time‐course of disease development and the complex nature of pathological processes in the brain attest to the difficulty of elucidating the molecular and cellular mechanisms underlying AD pathogenesis. To promote further investigation, animal models will be critical if progress is to be made. However, some AD mouse models do not accurately or reproducibly reflect AD in humans, and might thus need to be re‐evaluated as suitable models. As highlighted here, neuroinflammation is an important process in the pathogenesis of AD, and contributes a rate‐limiting component that potentially links Aβ amyloidosis with neurodegeneration and neuronal cell death through tauopathy. However, the pathological roles of neuroinflammation based on the brain microenvironment, as well as that contributed by the whole‐body macroenvironment, remain elusive. Understanding glial cell interactions associated with AD and other neurodegenerative disorders according to the newly coined term “gliostasis” (homeostasis of glial cells) might serve as a promising starting point. Relevant animal models will again be necessary to initiate such studies, and will serve as a fundamental research tool to elucidate the pathogenetic mechanisms underlying AD development and to develop preventive or therapeutic interventions to combat the disease.

## Conflict of interest

T.S. and T.C.S. serve as the advisor and CEO, respectively, for RIKEN BIO Co. Ltd., which sublicenses animal models to for‐profit organizations, the profits from which are used for the identification of disease biomarkers.
